# The first survey of cryptococcal cells in bird droppings across Bloemfontein, South Africa

**DOI:** 10.14202/vetworld.2021.2739-2744

**Published:** 2021-10-25

**Authors:** Gloria Kankam, Byron Christians, Maphori Maliehe, Nozethu Mjokane, Adepemi O. Ogundeji, Olufemi S. Folorunso, Carolina H. Pohl, Olihile M. Sebolai

**Affiliations:** Department of Microbiology and Biochemistry, University of the Free State, Bloemfontein, 9301, South Africa.

**Keywords:** Bloemfontein, Calcofluor-white stain, cryptococcal antigens, cryptococcal cells, cryptococcal meningitis, cryptococcal pneumonia, South Africa

## Abstract

**Background and Aim:**

Cryptococcal yeast cells are spread across different ecosystems through bird movement and are deposited in bird guano. These cells may be inhaled by humans and lead to cryptococcal pneumonia. In individuals with reduced immune T-cell populations, cells may disseminate to the brain and cause the often-deadly cryptococcal meningitis. In this study, we surveyed cryptococcal cells in bird droppings across the city of Bloemfontein, South Africa.

**Materials and Methods:**

We aseptically collected 120 bird dropping samples from 15 representative city sites. In the laboratory, samples were assessed with regards to location, weighed, and standardized to a mass of 1 g before suspension in 10 mL phosphate buffer saline. Samples were first screened usingCalcofluor-white stain as it is a rapid technique for the detection of fungi via binding to cell wall components such as chitin. After this, positive Calcofluor samples were serologically assayed for the cryptococcal antigen (CrAg). To confirm assay data, CrAg positive samples were then cultured on bird seed agar and resulting colonies were assessed using Indian ink.

**Results:**

We determined that 10/15 locations were positive for the CrAg. Pathogenic cells were identified on bird seed agar as brown colonies. When examined using microscopy, brown colony cells exhibited characteristic thick capsules representative of cryptococcal cells.

**Conclusion:**

This is the first proximate analysis showing the ecological distribution of cryptococcal cells in Bloemfontein. This is important as associated infections are acquired from the environment. Similarly, given the threat posed by cryptococcal cells to immunocompromised individuals, local authorities must initiate measures curbing the spread of these cells.

## Introduction

Several species exist within the *Cryptococcus* yeast genus; however, *Cryptococcus* *neoformans* and *Cryptococcus* *gattii* are of medical significance [1]. Both are characterized by a thick polysaccharide capsule surrounding cells, melanin production, urease activity, and the ability to grow at 37-42°C [1,2]. Importantly, these traits confer yeast pathogenicity and also distinguish them from other yeasts.

Birds are important cryptococcal cell dispersion vectors as their seasonal migration behaviors distribute cells across vast geographical spaces [3,4]. Cells predominantly inhabit the bird gut due to relatively low levels of bacterial competition, low pH, and an internal temperature of 42°C. Then, when cells are deposited in different environments as guano, they can become wind-swept and form bioairsolised infectious propagules, that is, desiccated yeast cells or basidiospores that germinate into yeast cells. As humans cannot control breath composition, these propagules can reach the lungs despite ciliary action and airway disturbance [5]. On reaching the lung space, propagules can cause cryptococcal pneumonia [6]. In susceptible individuals with immunosuppressive conditions such as human immunodeficiency virus/acquired immune deficiency syndrome (HIV/AIDS), the infection can spread to other body systems, especially the central nervous system [7]. Global molecular epidemiology studies have reported that *C. neoformans* *sensu lato* is the main etiological cryptococcosis agent in patients with HIV/AIDS, while *C. gattii sensu lato* primarily manifests as infections in immunocompetent individuals [8]. Globally, it has been reported that approximately 0.22 million cryptococcal meningitis cases occur annually, resulting in 180000 deaths everyyear [9]. Naicker *et al*. [10] reported that in South Africa, cryptococcal species were a more common cause of meningitis in adults than *Mycobacterium* *tuberculosis*, accounting for 62% of microbiologically confirmed cases. Furthermore, Naicker [11] reported a high genetic diversity in strains isolated in southern Africa - with molecular type VNB (endemic in southern Africa) being more virulent among *C. neoformans* molecular types. The domestication of birds further enhances the prevalence of cryptococcal cells, especially *C*. *neoformans* [12]. It was previously estimated that more than 300 bird species are domesticated [13]. Moreover, untamed birds such as pigeons inhabit public spaces in town squares and parks, where they come into close contact with people. Thus, by inference, these factors suggest people come into contact with bird guano potentially containing cryptococcal cells.

Given the threat posed by these cells to individuals with HIV/AIDS, we assessed the prevalence of cryptococcal species in bird droppings across the city of Bloemfontein, as importantly, clinical isolates arise from theenvironment [14]. Notably, in South Africa, only three studies have reported cryptococcal cells from environmental sources [8,15,16].

## Materials and Methods

### Ethical approval

This study did not use any live animal subjects. Therefore, no institutional ethical approval was taken.

### Study period and location

**Figure-1:**
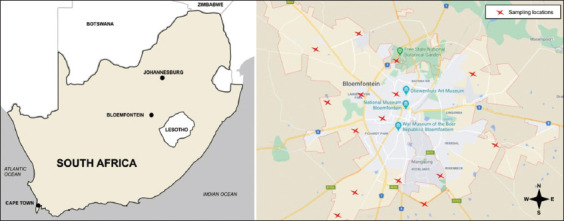
A map of South Africa (left) to assist the reader to locate the city of Bloemfontein within the boundaries of the country. A map of the city of Bloemfontein (right) showing the marked locations of sampling with red crosses. Hundred and twenty pigeon dropping samples were obtained from fifteen randomly chosen locations within Bloemfontein and its surrounding suburbs. The samples were taken to the pathogenic yeast laboratory for examination (Source: The image was prepared using CorelDraw and redrawn from Google Maps).

The study was conducted over 3 months (June to August 2017). Bloemfontein, as constituted by city and surrounding suburbs, was chosen as the sampling area, with 15 locations selected to represent different sampling sites (Figure-1). Sampling locations were chosen because they were frequented by birds, and contained old buildings and public spaces with a relatively high tree density. Bloemfontein contains approximately 520,000 people [17]. According to the South African state agency (Stats SA), approximately 13% of this population is HIV positive and is at greater risk of acquiring cryptococcal infections [18]. Other conditions such as cancer, diabetes, and organ transplantations are also threatened by cryptococcal infections [19].

### Sample collection and processing

We collected 120 samples by scooping droppings into sterile sealable plastic bags using sterile spatulas. These were labeled and immediately transported to the laboratory at the University of the Free State, Bloemfontein, South Africa. On arrival, samples from the same location were pooled and concentrated to 1 g standard weight, which resulted in 36 pooled samples (representative of the 15 locations) for testing. Samples were then suspended in 10 mL phosphate buffer saline (PBS; pH 7.4), vortexed for 1 min, and a 1:10 dilution was prepared to determine the presence of cryptococcal cells.

### Calcofluor-white staining

Samples were stained using a Calcofluor-white stain (Sigma-Aldrich, South Africa). Briefly, to 100 μL sample, 15 μL Calcofluor-white stains were added, and samples were incubated for 15 min in the dark at room temperature (22^o^C). Following the staining of cells with Calcofluor-white, a 100 μL sample was transferred to a black sterile microplate. Relative fluorescence units (RFUs) were then measured (exitation 360 nm; emission 460 nm) using a Fluoroskan plate reader (Thermo Fisher Scientific, USA) [20]. A PCR-grade water sample used to prepare PBS, was used as a negative control, while a *C*. *neoformans* H99 culture was used as a positive control.

### Cryptococcal antigen (CrAg) assay

Samples positive for Calcofluor-white staining were subjected to a CrAg immunoassay. We added 50 μL sample (from samples in PBS) to wells in an enzyme-linked immunosorbent assay (ELISA) plate (IMMY, USA) coated with antibodies specific for the CrAg. The ELISA plate was then treated according to manufacturer’s instructions, and absorbance readings were measured at 450 nm using a spectrophotometer (Biochrom EZ Read 800 Research, UK).

### Recovery of cells from positive samples

We next recovered cells from positive samples in the CrAg immunoassay by cultivation on bird seed agar [21]. Media were prepared according to Atlas and Snyder [22]. A 50 μL sample (from samples in PBS) was spotted onto the agar surface and a lawn createdusing a spread-plate method, *i*.e., a method of isolating and counting microbes in a test sample wherein the inoculum is spread evenly using the sterile L-shaped glass spreader over the surface of agar.Plates were then incubated at 30°C for 2 weeks, after which brown colonies (cell melanization) indicated growth of either *C. neoformans* or *C. gattii* [23]. A loop of brown colony cells was then Indian ink-stained, *i*.e., through using a flamed inoculation loop and sterile technique, cells are scooped from an agar plate and suspended (then mixed) in a drop of Indian ink on a clean microscope slide, and examined using an Olympus CKX53 microscope (Life Science, South Africa). For comparison purposes, *Candida albicans* SC5314 and *C. neoformans* H99 cultures were used as negative and positive controls, respectively.

## Results and Discussion

### Cryptococcal cells in bird droppings

**Figure-2:**
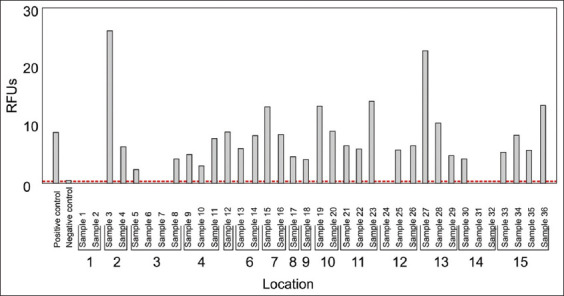
A graph showing the evidence of fungal life based on Calcofluor-white staining. The red line is the cutoff point. Values above the line are positive, and those that fall below the line are negative.

**Figure-3:**
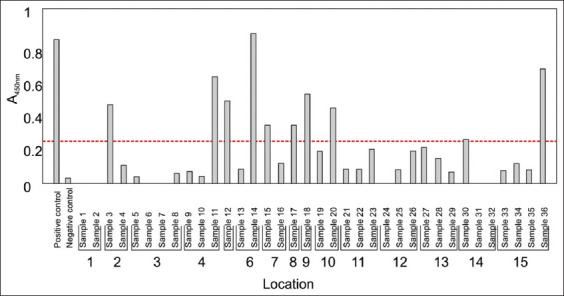
A graph showing the evidence of *Cryptococcus* species based on cryptococcal antigen immunoassay. The red line is the cut-off point. An absorbance reading below 0.265 is regarded as negative, while an absorbance reading of 0.265 and above is regarded as positive.

**Figure-4:**
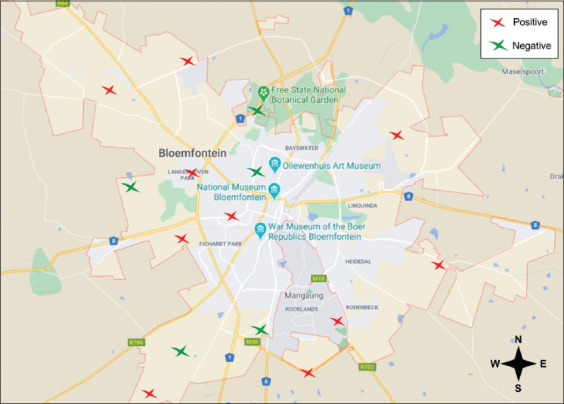
A map of Bloemfontein and surrounding suburbs showing the prevalence of *Cryptococcus* species in the different sampling locations (Source: The image was prepared using CorelDraw and redrawn from Google Maps).

The Calcofluor-white stain is a quick method that detects fungal cell wall components such as α-1,3 and β-1,4 glycosidic bonds in chitin and cellulose [24]. From our data, fungal species were identified in bird dropping samples (Figure-2). Readings from the negative control (water sample; RFUs = 0.6) were used as a threshold for chitin detection. Therefore, test samples with an RFUs reading below 0.6 (similarly to the water sample) were eliminated. Based on this approach, 29/36 samples provided readings above the threshold and were considered as harboring fungal species. These samples were then analyzed using a specific CrAg ELISA (Figure-3). Positive control data (included in the ELISA kit) were used as a threshold to detect the CrAg in samples. Finally, 10/36 samples tested positive for the CrAg. More importantly, these ten samples were obtained from ten different locations (from 15 sampling locations) across Bloemfontein (Figure-4). Thus, these findings indicated the presence of cryptococcal cells in Bloemfontein and provide the first approximation of fungal distribution across the city.

**Figure-5:**
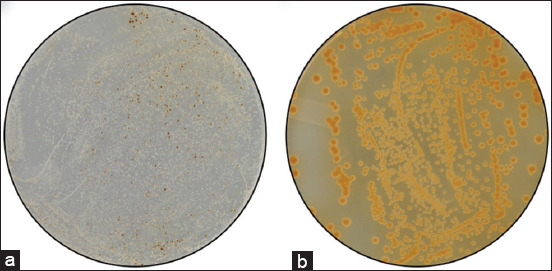
(a) depicts the growth of different organisms found in sample no. 17 (obtained from location 8) on a bird seed agar plate (b) was obtained after selecting a brown-pigmented colony on the plate (a) – this colony was spread on a sterile bird seed agar plate using the spread-plate method to yield a pure culture of cryptococcal colonies.

**Figure-6:**
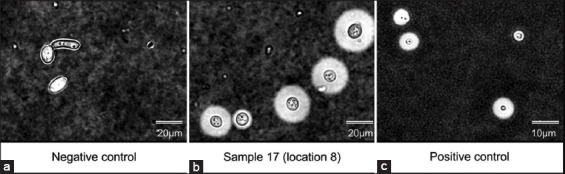
(a-c) The figure depicts India ink staining results obtained from a culture of *Candida albicans* SC5314 (negative control sample), *Cryptococcus neoformans* H99 (positive control sample), and a test sample (sample no. 17) obtained from location 8.

CrAg positive samples were cultivated on bird seed agar for the selective isolation of cryptococcal cells. A representative image (prepared using sample 17 from location 8) depicting cryptococcal brown colonies is shown (Figure-5). These colonies were generated due to interactions between cryptococcal phenoloxidase and caffeic acid (melanin-producing substrate) in Niger seeds (one of the ingredients found in bird food) [21]. According to the literature, *C. neoformans* or *C. gattii* colonies are best characterized and visualized on bird seed agar [25]. However, there are some limitations to the identification of these species on bird seed agar; for example, some strains do not produce pigmented colonies and may, therefore, be considered not to be *Cryptococcus* species (*i.e*., misdiagnosed as not *Cryptococcus* species). Moreover, bacterial contamination may slow down *Cryptococcus* species growth or prevent colony pigmentation; however, this can be limited and managed by adding streptomycin (20 mg/mL) to agar plates. Another limiting factor is that other fungal species such as *Wangiella* also form brown colonies on bird seed agar; however, this is not a result of an enzymatic reaction [26]. Our cultured samples were directly prepared from CrAg positive samples. According to Bottone negative staining is widely used to detect cryptococcal cells [27]. Indian ink is an ideal negative stain allowing for easily observable images against a dark background. The thick polysaccharide capsule surrounding *C. neoformans* and *C. gattii* generated a “halo effect” by Indian ink staining. A representative image (prepared using sample 17 from location 8) depicting the cryptococcal “halo effect” is shown in Figure-6.

At the time of the study, molecular techniques were unavailable; however, they will be adopted in subsequent work for a comprehensive molecular analysis of our samples. Nonetheless, for diagnostic purposes, the CrAg ELISA was perfectly adequate for determining cryptococcal cells in samples [28]. These cells also displayed the characteristic traits of cryptococcal species, including distinct morphology, capsule formation, and melanin production.

## Conclusion

*C. neoformans* species can spread rapidly due to constant bird migration. We demonstrated the presence of infectious *Cryptococcus* species across several sites in Bloemfontein. Importantly, a significant proportion of the immunocompromised population may be at risk of acquiring cryptococcal infections. Individuals must be mindful of domesticating birds, especially wild birds. Removing bird droppings from public spaces and increased sanitation of these areas may help minimize the threat of infection. Moreover, face masks and other personal protective equipment worn by municipal workers could limit the acquisition of occupation-associated infections.

## Authors’ Contributions

OMS: Conceptualization. GK: Conducted research in the laboratory, analyzed data, and wrote the first draft of the manuscript. BC, MM, NM, AOO, OSF, CHP, and OMS: Examined the data and critically revised the manuscript. All authors read and approved the final manuscript.
